# Anti-Inflammatory Activity of Oat Beta-Glucans in a Crohn’s Disease Model: Time- and Molar Mass-Dependent Effects

**DOI:** 10.3390/ijms22094485

**Published:** 2021-04-25

**Authors:** Ewa Żyła, Katarzyna Dziendzikowska, Dariusz Kamola, Jacek Wilczak, Rafał Sapierzyński, Joanna Harasym, Joanna Gromadzka-Ostrowska

**Affiliations:** 1 Department of Dietetics, Institute of Human Nutrition Sciences, Warsaw University of Life Sciences, Nowoursynowska 159c, 02-776 Warsaw, Poland; ewa_zyla@sggw.pl (E.Ż.); joanna_gromadzka_ostrowska@sggw.edu.pl (J.G.-O.); 2 Department of Physiological Sciences, Institute of Veterinary Medicine, Warsaw University of Life Sciences, Nowoursynowska 159, 02-776 Warsaw, Poland; dariusz_kamola@sggw.edu.pl (D.K.); jacek_wilczak@sggw.edu.pl (J.W.); 3 Department of Pathology and Veterinary Diagnostics, Institute of Veterinary Medicine, Warsaw University of Life Sciences, Nowoursynowska 159, 02-776 Warsaw, Poland; rafal_sapierzynski@sggw.edu.pl; 4 Adaptive Food Systems Accelerator—Research Centre, Wrocław University of Economics, Komandorska 118/120, 53-345 Wrocław, Poland; joanna.harasym@ue.wroc.pl; 5 Department of Biotechnology and Food Analysis, Wrocław University of Economics, Komandorska 118/120, 53-345 Wrocław, Poland

**Keywords:** oat beta-glucan, Crohn’s disease, cytokines, cytokine gene expression

## Abstract

Background: The incidence of Crohn’s disease (CD) is increasing worldwide, and it has currently become a serious public health issue in society. The treatment of CD continues throughout a patient’s lifetime, and therefore, it is necessary to develop new, effective treatment methods, including dietotherapy. The present study aimed to determine the effects of consumption of oat beta-glucans with different molar mass on colon inflammation (*colitis*) in the early stages of 2,4,6-trinitrobenzene sulfonic acid (TNBS)-induced CD in an animal model. Methods: Sprague–Dawley rats (control and TNBS-induced CD) were divided into three dietary groups and fed for 3 days (reflecting acute inflammation) or 7 days (reflecting remission) with a feed containing 1% low (βGl) or high (βGh) molar mass oat beta-glucan or a feed without this polysaccharide. The level of colon inflammatory markers and the expression of cytokines and their receptor genes were measured by ELISA and RT-PCR methods, respectively. Results: Acute inflammation or remission (3 or 7 days after TNBS administration, respectively) stages of experimentally induced CD were characterized by an increase in the level of inflammatory markers (IL-1, IL-6, IL-10, IL-12, TNF-α, CRP, MPO, COX, and PGE2) and the disruption of some cytokine signaling pathways as well as macro- and microscopic changes of colon tissue. The consumption of oat beta-glucans reduced the level of inflammatory markers and recovered the signaling pathways and histological changes, with stronger effects of βGl after 7 days of *colitis*. Conclusions: Dietary oat beta-glucans can reduce *colitis* at the molecular and organ level and accelerate CD remission.

## 1. Introduction

Inflammatory bowel disease (IBD), in which ulcerative *colitis* (UC) and Crohn’s disease (CD) are the two prevailing entities, is an important global public health concern with an increasing incidence in recent years [1,2]. IBD is a chronic idiopathic inflammatory disorder of the gastrointestinal tract, resulting from a combination of genetic predisposition, environmental factors, and inappropriate immune response to the gut microbiota [3].

Epidemiological data show that CD is mainly a disease occurring in developed countries, and the number of cases has been increasing rapidly in the last few decades [4,5]. The pathological process of CD begins in the mucosa, but over time, CD may spread to the intestinal wall along its entire surface. The disease is usually segmental, but inflammatory changes may affect the entire digestive tract, from the mouth to the rectum [6]. Chronic inflammation can result in complications such as fistulas, abscesses, or intestinal strictures. The course of CD is characterized by periods of exacerbation and remission. During exacerbation, there is an increased, abnormal stimulation of immune cells, which intensifies the synthesis and/or secretion of pro-inflammatory factors, e.g., cytokines such as TNF-α and Il-1. The main objectives of CD therapy are to reduce inflammation in the gut (induction of remission) and to prevent relapse (maintenance therapy). Another option is nutritional treatment during the early stages of CD by using plant-origin compounds with potent anti-inflammatory properties, similar to pharmacological treatment [7,8]. Such compounds include beta-glucans and other polysaccharides found in cereal grains.

Beta-glucans are a group of dietary fibers composed of D-glucose monomers linked by mixed 1,3; 1,4; or 1,6 β-glycosidic bonds; they are naturally found in the cell wall of fungi, yeast, seaweed, and higher plants such as cereals. Depending on the botanical origin, molar mass, spatial structure, and degree of purification, they can have different effects on the body [9]. Numerous studies have shown that beta-glucans of various origins can significantly stimulate several types of immune responses toward microorganisms and toxic and mutagenic factors [10,11,12,13].

To date, few research studies have investigated the health effects of oat beta-glucans with different molar mass, especially in the context of ongoing inflammatory mechanisms [14,15,16]. This prompted us to compare the effects of the pure fraction of low and high molar mass oat beta-glucans on colon inflammation in Sprague–Dawley rats as a model that corresponds to human CD. In the present study, we used highly purified oat 1,3/1,4-beta-D-glucan in two substantially different molar mass fractions. This study aimed to determine the molecular mechanisms involved during colon inflammation at the early stages of CD and during the administration of a diet containing oat beta-glucans with different molar mass.

## 2. Results

### 2.1. Feed Intake and Body Weight

**Figure 1:**
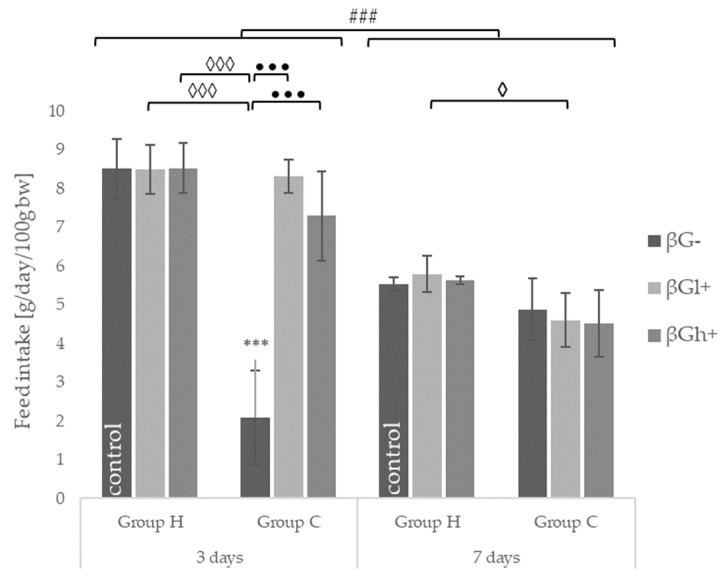
Feed intake 3 and 7 days after TNBS or 0.9% NaCl administration (mean ± SE) (n = 8 in each group). Significantly different from the control group *** *p* < 0.001; in the same experimental groups with regard to beta-glucans treatment ••• *p* < 0.001; between the experimental groups with regard to beta-glucans treatment ◊ *p* < 0.05, ◊◊◊ *p* < 0.001; in the same experimental groups with regard to beta-glucan treatment at different time points ### *p* < 0.001 (two-way ANOVA with Tukey’s post-hoc test).

Within 3 days after 0.9% NaCl or TNBS administration, the feed intake was at the similar level in control and *colitis* rats, except that the CβG- group that showed significantly lower feed consumption. The feed intake in CβGl+ and CβGh+ rats was significantly higher than that in CβG- rats. Feed intake 7 days after TNBS/NaCl administration regardless of the type of feed consumed was at the similar level (Figure 1).

The initial body weight of all 108 rats was similar, with a mean value of 414.0 ± 1.29 g. The final body weight after 3 days of TNBS or 0.9% NaCl administration decreased in all control and *colitis* groups, whereas after 7 days, the final body weight in all control groups increased slightly regardless of nutritional intervention from 8 g (HβGh+) or 14 g (HβG-) to 18 g (HβGl+) as compared to their initial body weight. The final body weight of rats with experimentally induced *colitis* 7 days after TNBS/NaCl administration was lower regardless of dietary regime (Table 1).

### 2.2. Macroscopic and Microscopic Changes

**Figure 2:**
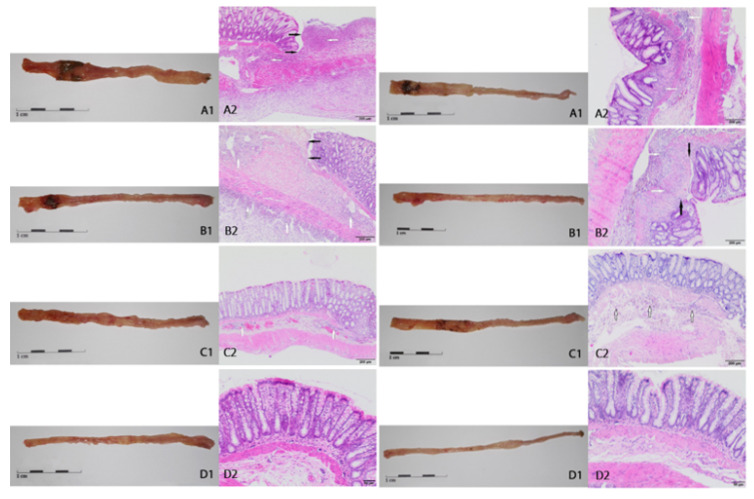
Macroscopic and microscopic changes in the descending colon 3 (**left side**) and 7 (**right side**) days after TNBS administration. (**A**1,**A**2): *colitis* CβG− groups; (**B**1,**B**2): *colitis* CβGl+ groups; (**C**1,**C**2): *colitis* CβGh+ groups; (**D**1,**D**2): health HCβG− groups. White arrows indicate multifocal/diffuse inflammatory infiltration of the mucosa and/or submucosa of varying severity, black arrows indicate margins of ulceration. Hematoxylin–eosin staining, magnification: (**B**2) left side and (**C**2) left side—4x; (**A**2) both sides, (**B**2) right side, (**C**2) right side—10x; (**D**2) both sides—20x. Various magnifications were used to better visualization of different lesions.

No macroscopic changes were found in the colon (score of mucosal damage = 0) in the control healthy rats without intestinal inflammation both after 3 or 7 days, regardless of the type of nutritional intervention or its absence. Macroscopic changes of colon mucosa from CβG- rats 3 and 7 days after TNBS administration confirmed severe local inflammation estimated as a median score of 3 or 2, respectively (Table 2), which was manifested by mucosal edema and minor bleeding (Figure 2(A1) left and right side, respectively). At the same time points in rats from the CβGl+ and CβGh+ groups, there was a reduction in the above-mentioned mucosal changes: median score = 2 in both cases (Table 2, Figure 2(B1,C1) left and right side, respectively).

Histological analysis of macroscopically altered colon sections confirmed extensive inflammation in the tissue of rats from the CβG- group (Figure 2(A2) left and right side). The lesions were trans-wall in nature, covering not only the mucosa but also the submucosal layer, which is a characteristic of CD. Microscopic changes included inflammatory changes together with infiltration of lymphocytes and granulocytes and focal or extensive ulceration, which was confirmed by a median score of 5 (3 days) or 3 (7 days) (Table 3, Figure 2(B2) left and right side, respectively). The consumption of feed supplemented with both beta-glucans did not reduce these changes after 7 days (Table 3, Figure 2(C2) right side), whereas after 3 days, the general microscopic score was significantly reduced in the CβGh+ group (Table 3, Figure 2(C2) left side). In the control groups, no microscopic damages in this part of the colon (after 3 days) or minor changes (after 7 days) were found (Table 3, Figure 2(D2) left and right side, respectively).

### 2.3. Levels of C-Reactive Protein and Selected Cytokines in the Colon Tissue

The levels of selected inflammatory markers were determined to describe the immune response of colon tissue to the induction of inflammation. Differences were observed in the effect of dietary intervention type (with two different fractions of oat beta-glucans) depending on the duration of the experiment (3 and 7 days after TNBS rectal administration).

Compared to the control group (HβG-), the induction of inflammation by the rectal administration of TBNS without nutritional intervention (group CβG-) at both time points (3 and 7 days) caused a significant increase in the levels of all pro-inflammatory cytokines (IL-1, IL-6, IL-12, and TNF-α) and C-reactive protein (CRP), whereas a decrease in the level of the anti-inflammatory cytokine IL-10 was observed. The increase in some pro-inflammatory cytokines (TNF-α, IL-1) and the decrease in IL-10 level in the colon wall were significantly greater at 3 days, whereas the increase in IL-12 was much higher at 7 days. The colon levels of CRP and IL-6 were comparable at 3 and 7 days. In the *colitis* groups, the consumption of feed containing oat beta-glucans resulted in a significant decrease in the level of all measured pro-inflammatory cytokines and an increase in the level of anti-inflammatory cytokine. Two-way ANOVA confirmed that these effects depended on both the molar mass of beta glucans and the length of the animal’s feeding period.

**Figure 3:**
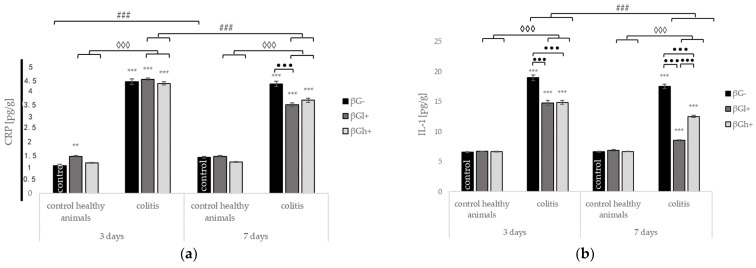

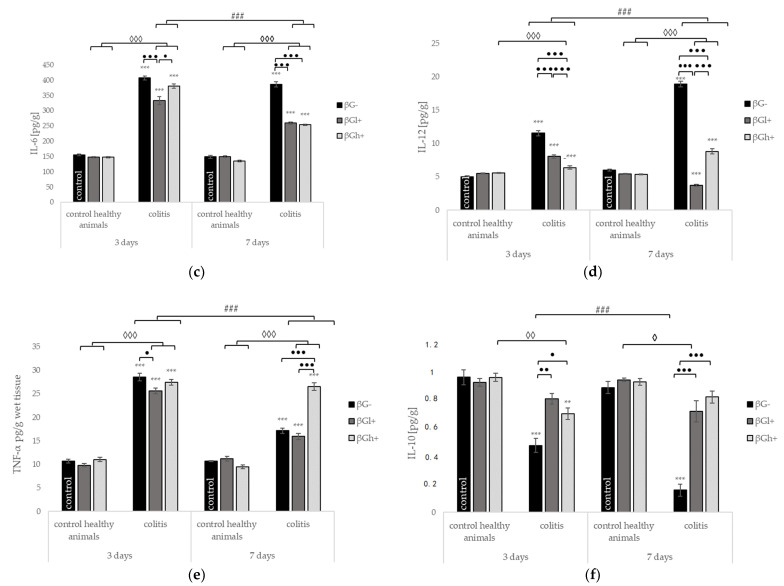
The effect of the development of *colitis* and dietary intervention on the level of C-reactive protein and cytokines in the colon tissue: (**a**) C-reactive protein (CRP); (**b**) interleukin 1 (IL-1); (**c**) interleukin 6 (IL-6); (**d**) interleukin 12 (IL-12); (**e**) tumor necrosis factor α (TNF-α) and (**f**) the interleukin 10 (IL-10) protein level (mean ± SE) (n = 8 in each group). Significantly different from the control group ** *p* < 0.01, *** *p* < 0.001; in the same experimental groups with regard to beta-glucans treatment • *p* < 0.05, •• *p* < 0.01, ••• *p* < 0.001; between the experimental groups with regard to beta-glucans treatment ◊ *p* < 0.05, ◊◊ *p* < 0.01, ◊◊◊ *p* < 0.001; in the same experimental groups with regard to beta-glucan treatment at different time points ### *p* < 0.001 (two-way ANOVA with Tukey’s post-hoc test).

The CRP level in the colon wall after 3 days of TNBS administration did not change significantly in the groups of animals consuming feed with the addition of both fractions of beta-glucans, while a significant reduction in the CRP level was found after 7 days, especially in animals from the CβGl+ group (Figure 3A). The IL-1 level was significantly reduced in the first period of *colitis* development (3 days) in both the CβGh+ and CβGl+ groups. In the next period of inflammation development (7 days), this reduction was significantly greater, especially in the CβGl+ group (Figure 3B). A significant reduction was also observed in the level of IL-6 in the colon wall at both time points, with the reduction being greater after 3 days of ongoing inflammation in the CβGl+ group. Seven days post TNBS administration, the CβGh+ and CβGl+ groups showed a similar level of this pro-inflammatory cytokine (Figure 3C). It was also found that TNF-α level measured 3 days after administration of TNBS was lower in group CβGl+, whereas 7 days after the induction of *colitis*, an increase in the TNF-α level was observed in the CβGh+ group (Figure 3D). The IL-12 level was also significantly decreased compared to that in animals, which did not receive beta-glucans in their feed, with a greater level of reduction at 7 days regardless of the molar mass of beta-glucan administered. In addition, the CβGh+ group and the CβGl+ group showed a greater reduction after 3 days and 7 days of ongoing inflammation, respectively (Figure 3E). Contrasting changes were found in the level of the anti-inflammatory cytokine IL-10, which increased significantly both after 3 and 7 days without a significant difference between the groups consuming feed with low or high molar mass beta-glucans (Figure 3F).

In the control groups, the levels of pro- and anti-inflammatory cytokines did not change significantly at both time points and in relation to the molar mass of beta-glucans.

### 2.4. Other Selected Inflammatory Markers

Additionally, we investigated the colon concentration of two enzymes associated with arachidonic acid transformation, which are involved in the inflammatory process: cyclooxygenase (COX) and myeloperoxidase (MPO). The levels of prostaglandin E2 (PGE2) and thromboxane A2 (TXA2) in the colon tissue were also analyzed.

The levels of COX, MPO, and PGE2 in the colon tissue significantly increased in animals with induced *colitis* fed with feed without beta-glucans (CβG- vs. HβG-) at both time points; however, a significantly greater increase in COX levels was found at 7 days, while a significantly greater increase in MPO and PGE levels was observed at 3 days after TNBS administration.

**Figure 4:**
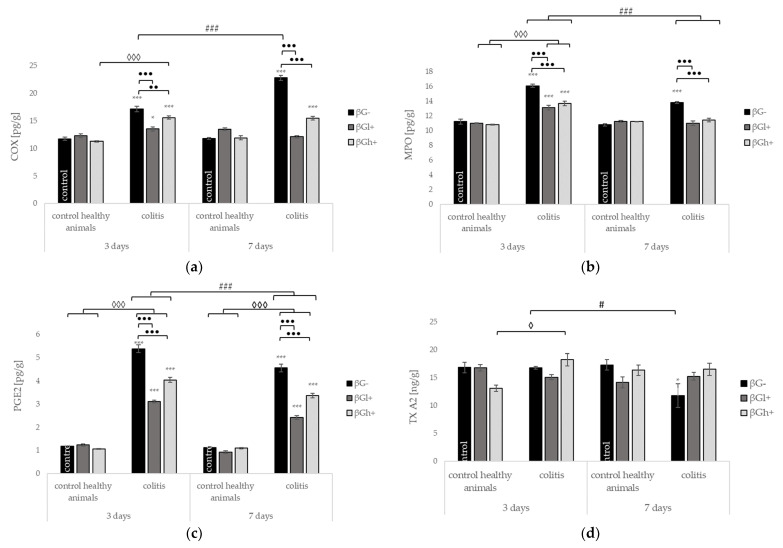
The effect of the development of *colitis* and dietary intervention on the level of selected immunological markers in the colon tissue: (**a**) total cyclooxygenase (COX); (**b**) myeloperoxidase (MPO); (**c**) prostaglandin E2 (PGE2); and (**d**) thromboxane A2 (TXA2) protein level (mean ± SE) (n = 8 in each group). Significantly different from the control group * *p* < 0.05, *** *p* < 0.001; in the same experimental groups with regard to beta-glucans treatment •• *p* < 0.01, ••• *p* < 0.001; between experimental groups with regard to beta-glucans treatment ◊ *p* < 0.05, ◊◊◊ *p* < 0.001; in the same experimental groups with regard to beta-glucan treatment at different time points # *p* < 0.05, ### *p* < 0.001 (two-way ANOVA with Tukey’s post-hoc test).

The levels of COX, MPO, and PGE2 were significantly decreased in *colitis* rats fed with feed containing beta-glucans compared to rats with *colitis* fed with feed without beta-glucans at both time points. Rats from the CβGl+ group showed a greater reduction in COX and PGE2 concentrations, rats from the CβGl+ and CβGh+ groups showed a greater reduction in MPO concentration (Figure 4a–c, respectively). The addition of low and high molar mass beta-glucans reduced the MPO level to the value detected in the control group (7CβGl+ and 7CβGh+ vs. 7HβG-). The consumption of feed with low molar mass beta-glucans also reduced the COX level in *colitis* rats to the value detected in the control group (7CβGl+ vs. 7HβG-). The TXA2 level in the colon did not significantly change at 3 days after TNBS administration, whereas at 7 days, the level of TXA2 significantly increased in the CβG- group as compared to that in the HβG- group. The consumption of feed containing both fractions of beta-glucans retained the TXA2 level in *colitis* rats to the value detected in the control group (Figure 4d).

In the control groups, the levels of COX, MPO, PGE2, and TXA2 did not change significantly depending on both the inflammation and the dietary beta-glucan fractions (Figure 4a–c).

### 2.5. Expression of Genes Encoding Inflammatory Cytokines and their Receptors

To elucidate the observed beneficial effects for both fractions of oat beta-glucans, the underlying molecular mechanisms were analyzed using the pathway-focused RT-PCR array for 50 different genes that encode inflammatory cytokines and their receptors expressed in the colon tissue. Our study also aimed to clarify whether the studied fractions of oat beta-glucans have different effects on the selected anti- and pro-inflammatory markers and whether this relationship is time-dependent.

The analysis was performed using the Rat Inflammatory Cytokines and Receptors array. The results showed that 3 days after TNBS administration, which corresponds to the acute *colitis* stage, of the 47 analyzed genes, 23 were up-regulated in the *colitis* group (CβG-) as compared to that in the healthy control group (HβG-). The up-regulated genes included those encoding interleukins and their receptors: *Il1a, Il1b, Il11, Il17a, Il17f, Il21, Il33, Il1r1, Il1rn, Il2rb, Il2rg, Il6r*, and *Il10ra*, and other inflammatory mediators*: Ifng, Lta, Ltb, Mif, Osm, Pf4, Spp1, Tnf, Tnfsf11*, and *Tnfsf14*. The supplementation of high molecular weight beta-glucan to the animal feed (CβGh+ group) up-regulated the expression of the *Il5, Il13,* and *Tnfsf10, Lta* and *Bmp2* genes and down-regulated the expression of the *Il1a, Il1b, Il11, Il17a, Il17b, Il17f, Il1r1, Il2rg, Il6r, Il10ra, Osm, Spp1, Tnf,* and *Tnfsf11* genes. The efficient *colitis* lowering effect was also observed in animals fed a diet supplemented with low molecular weight beta-glucan (CβGl+ group). Genes encoding *Ifng, Il3,* and *Il13* were up-regulated, whereas those encoding *Il1b, Il17a, Il21, Il5ra, Cd40lg, Osm, Tnf, Tnfsf11,* and *Tnfsf14* were down-regulated (Supplementary Materials, Table S1).

After 7 days of colitis induction, the expression of 18 genes, namely *Il1a, Il1b, Il3, Il4, Il11, Il17a, Il17f, Il1r1 Il1rn, Il2rb, Il10ra, Faslg, Ifng, Mif, Osm, Pf4, Spp1*, and *Tnfsf4*, was up-regulated whereas the expression of the *Bmp2, Cd40lg, Lta*, and *Tnfsf11* genes was down-regulated in the CβG- group as compared to that in the HβG- control group. In this stage of colitis, food intake with βGh (CβGh+ group) up-regulated the expression of the *Il13, Il21, Il27*, and *Lta* genes and down-regulated the expression of the *Il1a, Il11*, and *Il17a* genes. Moreover, the supplementation of low molecular weight beta-glucan to the animal feed (CβGl+ group) up-regulated the *Il21* and *Lta* genes expression and down-regulated the expression of the *Il3, Il11, Osm*, and *Spp1* genes (Supplementary Materials, Table S1).

## 3. Discussion

The prevalence of Crohn’s disease (CD) is increasing worldwide. CD is a chronic disease belonging to the IBD group and is characterized by alternating inflammation exacerbation and remission periods [17,18]. As a result of the complex and poorly understood etiopathogenesis of CD, it is essential to better understand the immune mechanisms related to this disease and to develop new, effective therapeutic options, including the use of natural and bioactive food compounds. Therefore, in this study, we analyzed the effects of oat beta-glucans administration at two time points, 3 and 7 days after TNBS administration. The use of the TNBS-induced *colitis* model allowed assessing immunological parameters at various stages of development of inflammatory lesions in the colon of rats, which corresponded to CD phases characterized by different severity levels in humans. *Colitis* induced by rectal administration of an ethanolic solution of TNBS, which is well described in the literature [19,20], causes transmural changes with clinical symptoms and a histological presentation characteristic of CD in humans [21,22]. This *colitis* model is also suitable for studying cell signaling pathways [23] and for analyzing a wide spectrum of cytokines [21,24]. Ethanol from the rectally administered solution is responsible for the damage of the colon epithelium and mucosal barrier breakdown. Ethanol allows the interaction of picrylsulfonic acid (TNBS) with the colon proteins, and consequently, it causes local necrosis resulting from ROS-induced oxidative damage. Both substances act synergistically to induce a strong local immune response, with the infiltration of the colon wall by lymphocytes and granulocytes and secretion of pro-inflammatory cytokines [21].

Three days after TNBS administration, rats fed the feed without oat beta-glucan supplementation developed local acute inflammation, which is a characteristic of the active phase of CD [23]. Histological evaluation of the colon confirmed extensive transmural inflammation, including inflammation of the mucosa and submucosal layers, which are characteristics of CD [22]. Seven days after TNBS administration, these symptoms improved, indicating the onset of inflammatory remission. Food intake and body weight gain decreased drastically after 3 days from TNBS administration and increased significantly after 7 days. Morphological changes indicated an improvement in animal health. Colon inflammation is also evidenced by a significant increase in the level of CRP, which is a marker of inflammation and is significantly elevated in patients in the exacerbation phase of CD [25]. The concentrations of pro-inflammatory cytokines (Il-1, Il-6, Il-12, and TNF-α) are also increased. The level of these cytokines increases in a time-dependent manner and confirms differences in the severity of *colitis* after 3 and 7 days of the ongoing process. The concentration of CRP and the cytokines Il-1 and Il-6 in the colon tissues were similar at both time points, which may be because the main signaling compound stimulating the synthesis of CRP is IL-6, while IL-1 also stimulates the synthesis of other acute-phase proteins [26]. Additionally, the increased protein level of pro-inflammatory cytokines, e.g., Il-1, Il-6, and TNF-α, causes a reduction in food intake as observed in our study and by Plata-Salaman (2001) [27].

After 7 days, rats with *colitis* had a significantly higher Il-12 level and a lower TNF-α protein level than those observed in control animals. The increase in IL-12 concentration after 7 days in animals fed control feed without beta-glucans may indicate the transition of the disease into a chronic form. This condition is a characteristic of both the TNBS-induced chronic inflammation model and the human CD, where the activity and proliferation of Th1 cells are increased due to an increase in IL-12 level [24]. In recent years, cytokine signaling pathways have been considered as a potential therapeutic target in IBD and in the treatment of autoimmune diseases such as CD [28,29]. Several attempts have been made to use monoclonal antibodies binding to the IL-12 subunit [30]. In the present study, activation of the immune response was confirmed both in terms of the protein concentration and gene expression level of inflammatory cytokines. Analysis of the expression of genes involved in cascading immune responses in the colon wall showed a significant up-regulation of genes encoding cytokines and their receptors. These changes were observed especially for pro-inflammatory cytokines, particularly in the IL-1 and TNF families in *colitis* animals fed with control feed without beta-glucans. The results are in line with the findings of other authors who also confirmed the increase in the concentration of pro-inflammatory cytokines in animals with exogenous *colitis* [31,32]. Additionally, all these signaling molecules are known to be important factors in the pathophysiology of CD [33,34]. In the present study, we observed a significant effect of both *colitis* induction and beta-glucan supplementation on the gene expression level of pro-inflammatory cytokines, including *Tnf, Il1a, Il1b, Il17a*, and *Il17b*. A three-day supplementation with low molar mass oat beta-glucans reduced the expression of the *Tnf* gene. *Tnf* gene expression was significantly up-regulated in rats with acute *colitis*, but it was decreased in rats from the CβGl+ group. Cui et al. (2005) conducted a study on 29 patients with IBD and 18 healthy patients and showed an 8-fold increase in the intestinal mucosa *TNF* gene expression level in patients with CD and a higher level of TNF in their feces [35]. An excess concentration of TNF reduces the protective abilities of the intestinal epithelium and enhances the processes of intestinal tissue fibrosis [36], which may contribute to the development of neoplastic diseases. Therefore, our results indicate that beta-glucans appear to be effective agents that can prevent *Tnf* overexpression and protect against cancerogenesis.

Our results show that the concentration of the anti-inflammatory cytokine IL-10 at both time points was lower in *colitis* rats than in the control rats, with higher levels observed after 7 days. The anti-inflammatory effect of IL-10 is related to the synthesis of IL-RAP, an antagonist of this protein, which inhibits the synthesis of many pro-inflammatory cytokines such as IL-1, IL-6, and TNF-α [37]. The cytokine Il-10 also plays an important role in the maintenance of overactive intestinal immune cells [38]. Patients with CD are reported to have normal or high levels of IL-10 [39]. Mutations in the *NOD2/CARD15* gene are associated with the development of CD [40,41]. The NOD2/CARD15 complex is an intracellular receptor for peptidoglycan, a component of the bacterial cell membrane, that activates the nuclear factor NF-κB and stimulates the MAPK protein kinase-related signaling pathway. The concentration of this protein increases along with the synthesis of pro-inflammatory cytokines TNF-α and IL-1β and other peptides with antibacterial activity. Under homeostatic conditions, the inflammatory response is inhibited by anti-inflammatory cytokines, including TGF-β and IL-10. In CD, mutations in the NOD2/CARD15 gene block these mechanisms, leading to persistent inflammation in the gut wall [42].

Feeding *colitis* rats with feed containing oat beta-glucans led to a reduction of high levels of CRP, Il-6, and Il-12 in the colon wall after 7 days. This finding indicates an accelerated remission. These observed changes were also more pronounced in rats fed with feed containing low molar mass beta-glucans. Our previous study showed that feeding rats for 21 days after TNBS administration with food containing beta-glucan with a low molar mass resulted in a more significant reduction in the concentration of pro-inflammatory cytokines and eicosanoids and stronger activation of immune cells than that observed in animals fed with food containing high molar mass beta-glucans [43]. In the present study, the consumption of feed with the addition of beta-glucans, regardless of their molar mass, resulted in change in Il-10 level in rats with *colitis* to that observed in the appropriate control animals; this finding suggests the stimulation of anti-inflammatory mechanisms by these polysaccharides.

Beta-glucans might regulate the impaired immune response, which is a characteristic of *colitis*, which has been proven in both in vitro and in vivo studies. The incubation of lipopolysaccharide (LPS)-stimulated human macrophages with a mixture of oat or barley beta-glucans has been shown to reduce the expression of pro-inflammatory cytokines such as IL-8, IL-1β, and IL-6 [44]. Additionally, the fungal beta-glucan inhibits dextran sulfate sodium (DSS)-induced ulcerative colitis and reduces the expression of inflammatory markers due to the suppression of the MAPK protein kinase signaling pathway [45]. Our previously published results have also shown that in rats with LPS-induced enteritis fed with feed containing low and high molar mass oat beta-glucan, the concentration of pro-inflammatory IL-1 and IL-12 was significantly reduced [14]. Liu et al. (2015) found a reduction in CD symptoms, including diarrhea and intestinal immune cell infiltration, as well as an increase in body weight gain and a reduction in inflammation in colitis rats fed with feed supplemented with oat beta-glucans. The proposed mechanism underlying this effect was based on beta-glucan-induced suppression of the IL-1β and IL-6 genes and nitric oxide synthase (iNOS) [46]. In our previous study, we found a normalization of IL-10 concentration in rats with enteritis after supplementation with oat beta-glucans [14]. Similar results were reported by Crespo et al. (2017) in an in vitro study. The authors found reduced IL-10 synthesis by macrophages isolated from the blood of rats fed with feed supplemented with 5% fungal beta-glucans [47]. Another study showed that the effect of beta-glucans on the concentration of IL-10 secreted by LPS-stimulated dendritic cells depends on the method of this polysaccharide preparation, its solubility, and degree of aggregation [48].

On the basis of our previous study and the results of other authors, it seems that oat beta-glucans can stimulate the secretion of anti-inflammatory cytokines. They simultaneously inhibit the secretion of pro-inflammatory cytokines. The immunostimulatory effect of beta-glucan intake occurs due to its ability to activate intestinal mucosa immune cells, which results from the binding of these polysaccharides to a specific membrane TLR and/or Dectin-1 receptors. The activation of both types of receptors is different in the acute and remission phase of CD. Dietary intake of βGl and βGh diminished colitis by the time-dependent modulation of autophagy and apoptosis, which involved TLRs (TLR4, TLR5) and Dectin-1 receptor activation, with βGI having a stronger effect on apoptosis (Caspase 3 expression) and βGh on autophagy (LC3B expression) [49]. Therefore, this might explain the difference in the effect of feeding with beta-glucans during 3 or 7 days after TNBS administration. Probably, the mechanism of the immunomodulatory effect of beta-glucans may also depend on its molar mass, which influences the severity of apoptosis and autophagy of colon epithelial cells. Our previous results show the most prominent reduction of mucosa and submucosa lymphocytes infiltration occurred after dietary supplementation with high molecular mass oat beta-glucan, while in the case of potential for the improvement of the cytokine gene and protein level, low molecular mass oat beta-glucan was more potent [43,49]. Beta-glucans with different molar mass activate different signaling pathways, and consequently, both forms reduce inflammation and accelerate remission, including restoration of integrity of the intestinal barrier [49]. High molar mass fractions of beta-glucan are known to form viscous gel-like dispersion with high adhesion properties. Long chains of glucose homopolymers coordinate water and reorganize charged side-branches of polymer resulting in a sticky surface easily bonding to any moisturized layer. By trapping beta-glucan linkages inside the gel, high molar mass fractions only tend to interact on a physical basis. Therefore, it is expected for feed containing high molar mass fractions of beta-glucan to adhere to the intestinal walls covering it. It can be expected that high molecular mass beta-glucan forms a protective coating on the internal intestinal wall, which improved tissue recovery potential and reduce the risk of secondary microbial infection. Meanwhile, the low molar mass fraction of beta-glucan forms light solutions where short chains are well distributed and dispersed, and due to low viscosity, beta-glucan is accessible for receptors to be reached. Once reaching and complementing the receptor, the bonded beta-glucan short polymeric chain induces transmission on metabolic pathways. Our results confirmed this hypothesis and may suggest that different molar mass oat beta-glucans could decrease inflammation by the downregulation of gene expression and the production of pro-inflammatory cytokines and other inflammatory signaling molecules.

Our results suggest that oat beta-glucans can suppress inflammation by reducing gene expression, thereby reducing the secretion of pro-inflammatory cytokines and other inflammatory signaling molecules. Moreover, they have the ability to restore the balance between the levels of pro-inflammatory and anti-inflammatory cytokines. Since the consumption of food supplemented with the addition of oat beta-glucans not only decreased the gene expression of pro-inflammatory cytokines but also decreased their concentration, the action of these polysaccharides is considered to occur at both tissue and molecular levels.

In this study, we found that the concentration of other inflammatory markers in the colon wall, including MPO, significantly increased as compared to that in healthy animals with higher concentration at 3 days after TNBS administration. This indicates an initial acute inflammation that might be associated with neutrophil infiltration into the colon mucosa. A reduction in inflammation was achieved at 7 days after TNBS administration. Other studies have reported that the active phase of IBD shows a higher concentration of MPO than that observed in the remission phase [50]. Higher MPO concentrations are reported in patients with more severe forms of IBD [51] and in patients with other inflammatory conditions [52,53]. Feeding animals with feed containing beta-glucans, regardless of their molar mass, reduced the colon concentration of MPO and accelerated remission of colitis. Furthermore, we found that the COX and PGE2 concentration increased in colitis rats fed with the control feed. Three and 7 days after TNBS administration, rats fed with feed containing both low and high molar mass beta-glucan showed a significant reduction in the concentration of these two proteins, with the low molar mass beta-glucan exhibiting greater effectiveness.

Our results confirmed the strong immunomodulatory properties of chemically pure oat beta-glucan obtained by alkaline extraction from ground oat bran [54]. These formulations seem to be as strong as the beta-glucan formulations obtained from various fungal species, including yeast, which are already used in IBD treatment [9,55,56,57]. This hypothesis is confirmed by the results of the use of fungal beta-glucans as an “adjuvant”, which strengthens the immune system of individuals with immune deficiencies or of patients undergoing anticancer therapy [58,59]. Oat beta-glucans have also been found to exhibit the same strong anti-tumor properties with cytotoxic effects on different human cancer cell lines [60,61]. Moreover, beta-glucans derived from oat induced trained immunity in monocytes and macrophages [62]. Our results showed that beta-glucan with a low molar mass has a particularly strong effect, which significantly accelerates the remission of CD and reduces the severity of inflammation at the later stage of its development. The unique immunomodulatory properties of oat beta-glucans, particularly those of low molar mass, point to the possibility of using them as a bioactive ingredient for special medical purposes for people suffering from inflammatory bowel disease, especially CD. Despite the promising results, more research is needed to evaluate its effect on human beings effectively.

## 4. Materials and Methods

### 4.1. Preparation of Deproteinated Oat Beta-Glucan Fractions

High- and low-molar mass beta-glucan preparations were obtained from beta-glucan enriched oat fiber with methods described elsewhere [63]. Both high and low molar mass preparations were obtained due to patented methods [63,64]. Beta-glucan was isolated with alkaline water (pH = 8.5 M NaOH) and then deproteinated at an isoelectric point (pH = 4.5), the protein precipitate was removed by centrifugation as described elsewhere [64]. Further purification was conducted using the enzymatic treatment of a group of proteolytic, peptidolytic, and amylolytic enzymes followed by subsequent enzyme protein precipitation at isoelectric points. Beta-glucan preparations purity was controlled with AOAC method 995.16 (Megazyme, Bray, Ireland). The molar mass of fractions was determined from intrinsic viscosity measurements of beta-glucan solutions and Mark–Houwink equation application. The proteinaceous matter removal was controlled with SDS PAGE, Kjeldahl, and Lowry methods. The purity (Table 4) of high molar mass (1.7 × 10^6^ ± 0.05 × 10^6^ g/mol) beta-glucan was 97.4 ± 0.7%, and the purity of low molar mass (5.9 × 10^4^ ± 0.3 × 10^4^ g/mol) beta-glucan was 99.1 ± 0.3%. Both fractions of these polysaccharides were used as a 1% addition to the semisynthetic rat’s feed.

The beta-glucan fraction of low molar mass revealed high beta-glucan content with a neglible amount of soluble proteins and amine nitrogen. In addition, antioxidant activity was very low and within the range of interference caused by residual proteins, which means that no residual polyphenolic compounds were present in the fraction. The high molar mass beta-glucan fraction was characterized by a slightly higher TPC value, which was in accordance with higher soluble protein content; however, both values were still in very low and almost neglible levels. Such characteristics suggest quite different mechanisms of antioxidative activity of both beta-glucan fractions, which was observed in vivo.

### 4.2. Animals and Experimental Design

The experiment was performed on adult male Sprague–Dawley rats (n = 96) purchased from Charles River Laboratories (Charles River, Sulzfeld, Germany). Experimental details, animal’s house conditions, and composition of experimental feeds were described in our previous paper [43].

**Figure 5:**
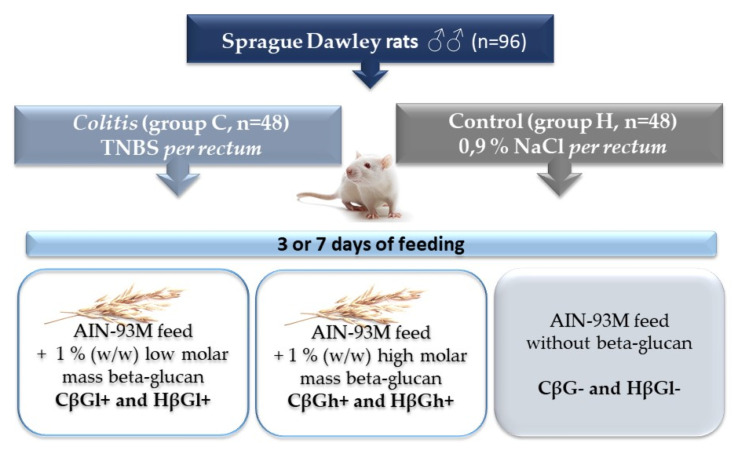
Scheme of experimental design of the study. TNBS: 2,4,6,-trinitrobenzenesulfonic acid alcohol solution.

Briefly, in colitis groups (C, n = 48) local colon inflammation was induced by TNBS (2,4,6,6-trinitrobenzenesulfonic acid) ethanol solution rectal administration, whereas in control groups (H, n = 48) (shame-operated), the same volume of 0.9% NaCl was given. Then, rats from C and H groups were divided into 3 nutritional subgroups (n = 8 each) which received for 3 or 7 days after TNBS/NaCl administration 3 types of feed differing in the addition of the oat beta-glucan fraction according to the group symbols below: CβGl+ and HβGl+ received for 3 or 7 days AIN-93M feed with 1% (*w/w*) low molar mass beta-glucan; CβGh+ and HβGh+ received for 3 or 7 days AIN-93M feed with 1% (*w/w*) high molar mass beta-glucan; CβG- and HβGl- received for 3 or 7 days AIN-93M feed without beta-glucan Figure 5. Feed intake was measured every 2 days by calculating the standardized intake (g/day/100 g rat body weight); final body weight was measured 12 h before euthanasia. After 3 or 7 days of feeding, rats were introduced into deep anesthesia (Isoflurane inhalation), bled from the heart, and then, the large intestine was removed to determined the stage of inflammation. After assessing the location of macroscopic lesions in the large intestine, the damaged sections of the colon were excised, part of them were fixed in 4% buffered formaldehyde, and the remaining fragments were frozen in liquid nitrogen and stored in −80 °C until biochemical analysis. Details of tissue collection and preparation as well as morphological criteria (scores) of *colitis* severity and microscopic colonic tissues damage (scores) were described in our previous paper [43].

The animal experiment was conducted after the approval of the II Local Animal Care and Use Committee in Warsaw (Resolution # 60/2015). All the procedures will be designed and conducted according to Polish and EU law regulations and with respect to 3R rules (Replacement, Reduction, and Refinement).

### 4.3. Evaluation of Histopathological Changes in the Wall of the Colon

The collected macroscopically altered colon specimens after being fixed in 4% buffered formaldehyde were processed via standard protocols (dehydration, clearing, and paraffinization). Sections from each paraffin block were cut in 4 µm thickness and stained with hematoxylin–eosin. The blinded slides were examined by a pathologist and the degree, extent, and type of inflammation, ulceration, and degree of regeneration/architectural distortion were assessed. Finally, microscopic score according to Galvez et al. (2001) was established for all rats [65].

### 4.4. Determination of Inflammation Parameters

The phosphate-buffered saline was used to homogenize colon tissue samples. In tissue homogenates, the concentration of pro- and anti-inflammatory cytokines (IL-1, IL-6, IL-10, and IL-12), tumor necrosis factor alpha (TNF-α), C-reactive protein (CRP), prostaglandin E2 (PGE2), and thromboxane A2 (TXA2) were determined using a competitive specific enzyme immunoassay (ELISA) according to the manufacturer’s instructions (DRG Instruments GmbH, Marburg, Germany). In turn, the concentration of total cyclooxygenase (COX) and myeloperoxidase activity (MPO) were determined by enzymatic assay (Cayman Chemical, Ann Arbor, MI, USA).

### 4.5. RNA Isolation, Reverse Transcription, and Real-Time PCR

RNA isolation, reverse transcription, and real-time PCR methods were described prviously by Żyła et al. (2019) [43]. Briefly, total RNA from the colon samples was isolated using the RNeasy Lipid Tissue Mini Kit (Qiagen, Hilden, Germany) according to the manufacturer’s instruction. The RNA concentration and purity were measured using a NanoDrop™ 2000 spectrophotometer (Thermo Fisher Scientific, Waltham, MA, USA) that indicated RNA concentration and purity. The RNA integrity was assessed using an Agilent Bioanalyzer 2100 system with the RNA 6000 Nano LabChip^®^ kit (Agilent Technologies, Palo Alto, CA, USA); minimal acceptable RNA integrity (RIN) was 9. Then, RNA was converted using an RT2 First Strand Kit (Qiagen, Hilden, Germany) to complementary DNA (cDNA) and prepared by RT-PCR assay according to the manufacturer’s protocol. The expression profiling was performed using the RT² Profiler™ PCR Rat Inflammatory Cytokines and Receptors array (Qiagen, Hilden, Germany) based on the manufacturer’s instructions, in one technical replicates for each colon sample. PCR reactions, with an initial 10-min step at 95 °C followed by 40 cycles of 95 °C for 15 s and 60 °C for 1 min, were carried out on a Stratagene Mx3005P qPCR system (Agilent Technologies, Palo Alto, CA, USA). Relative gene expression was calculated using the ΔΔCt method with Ldha and Rplp1 as housekeeping genes using the Data Analysis Qiagen Center (Qiagen, Hilden, Germany). The results are shown as the relative gene expression of the target vs. reference gene in relation to the healthy control group (HβG-) calculated as 1.

### 4.6. Statistical Analysis

Results data are represented as mean ± SE. Statistical analyses were performed with Statistica software version 13.0 (StatSoft Inc.,Tulsa, OK, USA). Using a two-way analysis of variance (ANOVA), we compared differences between groups, which was then followed by a post hoc Tukey’s test. Differences were considered as significant at *p* < 0.05, *p* < 0.01, *p* < 0.001.

## 5. Conclusions

Our results indicate that the therapeutic effect of oat beta-glucans depends on their molar mass and stage of CD. The consumption of low molar mass beta-glucans reduce the gene expression of pro-inflammatory cytokines and their protein concentration in the colon, suggesting effects at both the tissue and molecular levels, which is a good prognostic for use in CD dietotherapy. Meanwhile, high molecular mass beta-glucan, due to its higher viscosity improved the tissue recovery potential that results in apparently better histological response. The neutrality of beta-glucan in the normal colon and high activity in colitis prove that this substance is safe for the organism.

## Figures and Tables

**Table 1:** Final body weight (mean ± SE) (n = 8 in each group).

Time (days)	Healthy Group			*Colitis* Group		
	HβG−	HβGl+	HβGh+	CβG−	CβGl+	CβGh+
3	327.63 ± 6.64	326.38 ± 4.51	333.25 ± 3.65	304.5 ± 9.22	322.13 ± 9.86	317.5 ± 8.32
7	427.75 ± 5.14	431.5 ± 5.43	422.0 ± 3.96	406.25 ± 19.48	403.5 ± 11.5	397.88 ± 7.48

**Table 2:** Score of macroscopic damages of the colon mucosa. Damage score (0–4). Data are expressed as medians (n = 8 in each group).

Time (days)	Healthy Group			*Colitis* Group		
	HβG−	HβGl+	HβGh+	CβG−	CβGl+	CβGh+
3	0	0	0	3	2	2
7	0	0	0	2	2	2

**Table 3:** Score of microscopic damages of the colon mucosa and submucosa. Damage score (0–5). Data are expressed as medians (n = 8 in each group).

Time (days)	Healthy Group			*Colitis* Group		
	HβG−	HβGl+	HβGh+	CβG−	CβGl+	CβGh+
3	0	0.5	0.5	5	5	3.5
7	1	1	1	3	3	3

**Table 4:** Physicochemical characteristics of beta-glucan fractions.

BG Fraction	Molar Mass (g/mol)	Purity (%)	TPC (GAE/ 1 g d.b)	DPPH (μmol of Trolox)	Soluble Proteins (%/db)	Nitrogen × 5.83 (%/db)
low	5.9 × 10^4^ ± 0.3 × 10^4^	99.1 ± 0.3	0.20	5.41	0.09 ± 0.01	1.14 ± 0.14
high	1.7 × 10^6^ ± 0.05 × 10^6^	97.4 ± 0.7	0.48	6.03	0.70 ± 0.14	1.17 ± 0.08

## Data Availability

The data that support the findings of this study are available on request from the corresponding author [K.D.].

## Supplementary Materials

The following are available online at https://www.mdpi.com/article/10.3390/ijms22094485/s1. Table S1: Changes in gene expression 3 and 7 days after TNBS administration. Regulation of genes encode inflammatory cytokines and their receptors in the colon tissue. Results are reported as fold regulation > 2; bold means statistical significance in Student’s *t*-test *p* ≤ 0.05.
