# Supplementary material for: Anti-Inflammatory Activity of Oat Beta-Glucans in a Crohn’s Disease Model: Time- and Molar Mass-Dependent Effects
Source: Int J Mol Sci. 2021 Apr 25;22(9):4485. doi: 10.3390/ijms22094485 (PMC8123447; doi:10.3390/ijms22094485)
Supplement: Supplementary file 1 [file ijms-22-04485-s001.zip › ijms-1188864-supplementary.pdf]

		CβG- vs HβG-	CβGl+ vs CβG-	CβGh+ vs CβG-	HβGl+ vs HβG-	HβGh+ vs HβG-
3 days	↑	**Σ 23 gens**<br>IL and ILR: Il1a, Il1b, Il11, **Il17a**, Il17f, Il21, Il33, Il1r1, Il1rn, Il2rb, Il2rg, **Il6r**, **Il10ra**<br>Other: Ifng, Lta, Ltb, **Mif**, Osm, **Pf4**, **Spp1**, **Tnf**, Tnfsf11, Tnfsf14	**Σ 3 gens**<br>IL: Il3, Il13<br>Other: Ifng	**Σ 5 gens**<br>IL : Il5, Il13<br>Other: **Bmp2**, Lta, **Tnfsf10**	**Σ 6 gens**<br>ILR: **Il2rg**, Il6r, Il10ra<br>Other: Cd40lg, Lta, Tnfsf14	**Σ 9 gens**<br>IL and ILR: Il1a, Il2rg, Il10ra<br>Other: Cd40lg, Lta, Ltb, Tnf, Tnfsf11, Tnfsf14
	↓	**Σ 2 gens**<br>other: **Bmp2, Tnfsf10**	**Σ 9 gens**<br>IL and ILR: Il1b, Il17a, Il21, Il5ra<br>other: Cd40lg, Osm, Tnf, Tnfsf11, Tnfsf14	**Σ 14 gens**<br>IL and ILR: Il1a, Il1b, Il11, Il17a, Il17b, Il17f, Il1r1, Il1rn, Il2rg, **Il6r**<br>other: Osm, Spp1, **Tnf**, Tnfsf11		
7days	↑	**Σ 18 gens**<br>IL and ILR: Il1a, Il1b, Il3, Il4, Il11, Il17a, Il17f, Il1r1, Il1rn, **Il2rb**, Il10ra<br>Other: Faslg, Ifng, Mif, Osm, Pf4, Spp1, **Tnfsf4**	**Σ 2 gens**<br>IL: Il21<br>Other: Lta	**Σ 4 gens**<br>IL: Il13, Il21, Il27<br>Other: Lta	**Σ 6 gens**<br>IL: Il3, Il4, Il13, Il17a, Il27<br>Other: Ifng	**Σ 6 gens**<br>IL: Il3, Il15, Il17a, Il27<br>Other: Spp1, Tnfsf4
	↓	**Σ 4 gens**<br>Otrer: **Bmp2**, Cd40lg, Lta, Tnfsf11	**Σ 4 gens**<br>IL: Il3, Il11<br>Other: Osm, Spp1	**Σ 6 gens**<br>IL: Il1a , Il11, Il17f<br>Other: Osm, Pf4, Spp1		**Σ 8 gens**<br>ILR: Il1rn, Il2rg, Il10ra<br>Other: Cd40lg, Lta, Ltb, Osm, Tnfsf11

Changes in gene expresion 3 and 7 days after TNBS administration. Regulation of genes encode inflammatory cytokines and their receptors in the colon tissue: IL and LIR (interleukins and their receptors); Other (other inflammatory mediators). Results are reported as fold regulation > 2; bold means statistical significance in Student's t-test $p \leq 0.05$

Interleukins and their receptors: interleukin 1 alpha (*Il1a*), interleukin 1 beta (*Il1b*), interleukin 3 (*Il3*), interleukin 4 (*Il4*), interleukin 5 (*Il5*), interleukin 11 (*Il11*), interleukin 13 (*Il13*), interleukin 15 (*Il15*), interleukin 17A (*Il17a*), interleukin 17B (*Il17b*), interleukin 17F (*Il17f*), interleukin 21 (*Il21*), interleukin 27 (*Il27*), interleukin 33 (*Il33*), interleukin 1 receptor, type I (*Il1r1*), interleukin 1 receptor antagonist (*Il1rn*), interleukin 2 receptor, beta (*Il2rb*), interleukin 2 receptor, gamma (*Il2rg*), interleukin 5 receptor, alpha (*Il5ra*), interleukin 6 receptor (*Il6r*), interleukin 10 receptor, alpha (*Il10ra*)

Other inflammatory mediators: bone morphogenetic protein 2 (*Bmp2*), CD40 ligand (*Cd40lg*), Fas ligand (TNF superfamily, member 6) (*Faslg*), interferon gamma (*Ifng*), lymphotoxin alpha (TNF superfamily, member 1) (*Lta*), lymphotoxin beta (TNF superfamily, member 3) (*Ltb*), macrophage migration inhibitory factor (*Mif*), oncostatin M (*Osm*), platelet factor 4 G01 (*Pf4*), secreted phosphoprotein 1 (*Spp1*), tumor necrosis factor (TNF superfamily, member 2) (*Tnf*), tumor necrosis factor (ligand) superfamily, member 4 (*Tnfsf4*), tumor necrosis factor (ligand) superfamily, member 10 (*Tnfsf10*), tumor necrosis factor (ligand) superfamily, member 11 (*Tnfsf11*), tumor necrosis factor (ligand) superfamily, member 14 (*Tnfsf14*)
